# Walking and Social Reminiscence in Gentrifying Neighborhoods Among Older Black Adults: Feasibility and Outcomes

**DOI:** 10.1093/geroni/igaf122.295

**Published:** 2025-12-31

**Authors:** Raina Croff, Sophia Aron, Anne Wachana, Patrice Fuller, Nora Mattek, Juell Towns, Jeffrey Kaye

**Affiliations:** Oregon Health & Science University, Portland, Oregon, United States; Oregon Health & Science University, Portland, Oregon, United States; Oregon Health & Science University, Portland, Oregon, United States; Oregon Health & Science University, Portland, Oregon, United States; Oregon Health & Science University, Portland, Oregon, United States; University of Washington, Seattle, Washington, United States; Oregon Health & Science University, Portland, Oregon, United States

## Abstract

The Sharing History through Active Reminiscence and Photo-imagery (SHARP) study uses Black history amidst the erasure of gentrification and the disparate risk of memory loss to motivate healthier aging among older Black older adults. In two pilots, fourteen triads of healthy and mildly cognitively impaired participants walked 1-mile routes 3x/week over 24 weeks in Portland’s gentrifying historically Black neighborhoods. Using a group tablet, triads accessed a menu of 72 themed routes via the SHARP walking application. Along routes, GPS-linked historical images of local Black life and culture from 1940-2010 prompted conversational reminiscence. Walking narratives were recorded for a digital oral history archive. Retention was 74% and 86% for pilots, and 100% and 92%, respectively, were “extremely likely” to recommend SHARP to friends and family. Mean rank scores indicated program readiness with minor changes, effective conversational prompts, and appropriate pace and dose. Self-rated health, mood, activity levels, and energy improved, days feeling downhearted decreased, and days feeling calm/peaceful were maintained or improved. Among Cohort 2, cognitive assessment scores were maintained or improved for 67%; for MCI, 76% had mean improvement of 2.4 (p = .045). Blood pressure and weight decreased for 78% and 44%, respectively. Thirty focus groups were completed. Participants noted that the triadic structure created group accountability, motivated sustained engagement, curated friendships, and expanded social networks. Walking also increased physical and social activity outside the program, inspired healthier habits and self-care, improved awareness of cognitive decline risk and personal agency, and cultivated a deep-seated sense of community connection.

